# Envelope Interactions in Multi-Channel Amplitude Modulation Frequency Discrimination by Cochlear Implant Users

**DOI:** 10.1371/journal.pone.0139546

**Published:** 2015-10-02

**Authors:** John J. Galvin, Sandra I. Oba, Deniz Başkent, Monita Chatterjee, Qian-Jie Fu

**Affiliations:** 1 Department of Head and Neck Surgery, David Geffen School of Medicine, University of California Los Angeles, Los Angeles, California, United States of America; 2 Department of Otorhinolaryngology, Head and Neck Surgery, University Medical Center Groningen, University of Groningen, Groningen, The Netherlands; 3 Research School of Behavioral and Cognitive Neurosciences, Graduate School of Medical Sciences, University of Groningen, Groningen, The Netherlands; 4 Auditory Prostheses & Perception Lab, Boys Town National Research Hospital, Omaha, Nebraska, United States of America; University of Melbourne, AUSTRALIA

## Abstract

**Rationale:**

Previous cochlear implant (CI) studies have shown that single-channel amplitude modulation frequency discrimination (AMFD) can be improved when coherent modulation is delivered to additional channels. It is unclear whether the multi-channel advantage is due to increased loudness, multiple envelope representations, or to component channels with better temporal processing. Measuring envelope interference may shed light on how modulated channels can be combined.

**Methods:**

In this study, multi-channel AMFD was measured in CI subjects using a 3-alternative forced-choice, non-adaptive procedure (“which interval is different?”). For the reference stimulus, the reference AM (100 Hz) was delivered to all 3 channels. For the probe stimulus, the target AM (101, 102, 104, 108, 116, 132, 164, 228, or 256 Hz) was delivered to 1 of 3 channels, and the reference AM (100 Hz) delivered to the other 2 channels. The spacing between electrodes was varied to be wide or narrow to test different degrees of channel interaction.

**Results:**

Results showed that CI subjects were highly sensitive to interactions between the reference and target envelopes. However, performance was non-monotonic as a function of target AM frequency. For the wide spacing, there was significantly less envelope interaction when the target AM was delivered to the basal channel. For the narrow spacing, there was no effect of target AM channel. The present data were also compared to a related previous study in which the target AM was delivered to a single channel or to all 3 channels. AMFD was much better with multiple than with single channels whether the target AM was delivered to 1 of 3 or to all 3 channels. For very small differences between the reference and target AM frequencies (2–4 Hz), there was often greater sensitivity when the target AM was delivered to 1 of 3 channels versus all 3 channels, especially for narrowly spaced electrodes.

**Conclusions:**

Besides the increased loudness, the present results also suggest that multiple envelope representations may contribute to the multi-channel advantage observed in previous AMFD studies. The different patterns of results for the wide and narrow spacing suggest a peripheral contribution to multi-channel temporal processing. Because the effect of target AM frequency was non-monotonic in this study, adaptive procedures may not be suitable to measure AMFD thresholds with interfering envelopes. Envelope interactions among multiple channels may be quite complex, depending on the envelope information presented to each channel and the relative independence of the stimulated channels.

## Introduction

Given the limited spectral resolution of cochlear implants (CIs), temporal envelopes convey important speech cues for CI users. As such, CI users’ temporal processing capabilities may contribute to their speech understanding. Single-channel amplitude modulation detection (AMD) has been extensively measured in CI users [1–11] and has been correlated with CI users’ speech performance [3,12]. Similarly, CI users’ single-channel amplitude modulation frequency discrimination (AMFD) has been correlated with CI users’ prosody perception [13–15] and tonal language perception [16]. However, in everyday listening with clinical processors, CI users must process multiple temporal envelopes. Because of multi-channel loudness summation, current levels on individual channels must often be reduced in clinical processors to provide a comfortable operating range [17–19]. Single-channel AMD and AMFD have been shown to depend on current level [1–9,11,16]. At the same loudness, multi-channel AMD has been shown to be significantly poorer than single-channel AMD, due to the reduced current levels needed to compensate for multi-channel loudness summation [20]. However, at the same loudness, single- and multi-channel AMFD thresholds have not been shown to be significantly different [21], despite differences in current level. Previous studies have also shown that single-channel AMD thresholds can vary across stimulation site [6], though no clear effect of across-site variability has been shown for multi-channel AMD [20]. For AMFD, It is unclear how across-site variability may affect multi-channel perception. Thus, many factors may contribute to CI users’ multi-channel temporal envelope processing: listening task (envelope detection vs. envelope frequency discrimination), current level, multi-channel loudness summation, across-site differences in temporal processing, etc.

One issue when measuring AMD is the contribution of potential loudness cues associated with amplitude modulated (AM) stimuli [22]. As such, it is unclear whether AMD represents CI users’ temporal processing limits or their sensitivity to loudness cues in AM stimuli. While there are methods to limit the contribution of potential AM loudness cues [10,23], such current level adjustments and/or roving may introduce too much variability in AMD thresholds. As such, discrimination of AM frequency, rather than detection of AM, may better represent temporal processing limits of CI users. AMFD is typically measured using AM depths that are well above AMD threshold. Loudness differences across AM frequency are inconsistent and typically small [24]. Accordingly, less current level compensation and jitter is needed when measuring AMFD than for AMD, resulting in a potentially less “noisy” measure of CI users’ temporal processing.

AMFD has been shown to be better when the target AM was delivered to multiple channels than to any of the single component channels used for the multi-channel stimuli [21,25]. As noted above, when single- and multi-channel stimuli are similarly loud and at a comfortably loud presentation level, no significant difference in AMFD was observed [21]. It is unclear how across-site variability might contribute to the multi-channel advantage in AMFD. When single-channel AMFD was measured at summation-adjusted current levels, performance was near chance-level [21], obscuring across-site differences in performance. Thus, when multi-channel loudness summation is considered, it may be difficult to observe how channels are combined when discriminating coherent AM delivered to multiple channels.

Many previous studies have explored how competing envelopes may interfere with CI users’ ability to detect or discriminate target AM. For AMD, significant amounts of “envelope masking” (the difference in AMD threshold between a modulated and steady state masker) have been observed even when the target AM channel is spatially remote from the masker channel [26–27]. As such, central processes are thought to contribute strongly to CI users’ temporal envelope perception. Similarly, AMFD thresholds have been shown to be greatly elevated in the presence of competing envelope information, even when the target and masker channels are spatially remote [28–29]. In general, CI users seem unable to segregate even large AM frequency differences between the target and masker channel. In these previous studies, presentation levels for the target AM channel were relatively high, thus ensuring good baseline single-channel AMD or AMFD thresholds. Also in these studies, there was typically no adjustment for multi-channel loudness summation. Because the multi-channel stimuli only contained 2 channels, and because of the relatively high presentation levels, multi-channel loudness summation would not be expected to significantly contribute to the pattern of results observed. However, when a larger number of channels are considered along with the attendant loudness summation, baseline single-channel thresholds at summation-adjusted levels would most likely be much poorer than observed in previous AMD or AMFD studies. Indeed, at summation adjusted levels, single-channel AMFD was recently shown to be at near chance-level [21]. And while widely spaced channels have been used in some previous studies [28–29], there have been few comparisons of envelope interference between widely and narrowly spaced channels. If interference were to occur at the edges of the spread of excitation from multiple channels, less interference would be expected for widely spaced electrodes. At reduced summation-adjusted current levels, the spread of excitation would be less broad [30–31], which might reduce channel interaction, especially for widely spaced channels. In these previous studies, it is also unclear how across-site differences in temporal processing may have contributed to the degree of interference between the masker and target channels, as temporal processing was not typically measured for masker channels. One previous AMD study showed no clear relationship between the envelope sensitivity of the masker channel and the amount of envelope masking produced by the masker channel [27].

Taken together, results from these previous studies suggest that multi-channel envelope perception may affected by the information in each channel (coherent or competing AM), multi-channel loudness summation, across-site difference in temporal processing, and the spatial overlap in the spread of excitation from each component channel. In this study, AMFD was measured using multi-channel stimuli in which the target AM was delivered to 1 of 3 channels and the reference AM was delivered to the other 2 channels. The component channels were either widely or narrowly spaced to explore different degrees of channel interaction. The target AM channel was varied to explore across-site differences in temporal processing. To examine how AM discrimination was affected by the type of envelope information delivered to multiple channels, the present data were compared to those from a previous related study in which the target AM was delivered to a single channel or to all 3 channels [21]. In all cases, whether with single or multiple channels, AMFD data was compared using summation-adjusted current levels to explore temporal processing at the reduced current levels that might be used in clinical processors. Comparing AMFD with single and multiple channels at the same summation adjusted current levels provided an opportunity to examine the effects of loudness and the type of information delivered to each channel on AM discrimination.

## Methods

### Subjects

Five adult, post-lingually deafened CI users participated in this study. All were users of Cochlear Corp. devices and all had more than 2 years of experience with their implant device. Relevant subject details are shown in Table 1. All 5 subjects previously participated in a related AMFD study [21].

**Table 1:** CI subject demographics.

Subject	Age at testing (yrs)	Age at implantation (yrs)	Duration of deafness (yrs)	Etiology	Device	Strategy
**S1**	70	60	23	Genetic	N24	ACE
**S2**	79	77	35	Otosclerosis	N5	ACE
**S3**	28	26	11	Acoustic Neuroma	Freedom	ACE
**S4**	67	59	20	Meniere’s/ Otosclerosis	Freedom	ACE
**S5**	78	76	8	Unknown	N5	ACE

N24 = Nucleus 24; N5 = Nucleus 5; ACE = Advanced combination encoder

### Ethics Statement

All subjects provided written informed consent prior to participating in the study, in accordance with the guidelines of the St. Vincent Medical Center Institutional Review Board (Los Angeles, CA), which specifically approved this study. All subjects were financially compensated for their participation.

### Stimuli

Stimuli were similar to those used in a previous related study [21]. All stimuli were 300-ms biphasic pulse trains; the stimulation mode was monopolar, the stimulation rate was 2000 pulses per second (pps) per electrode, the pulse phase duration was 25 μs and the inter-phase gap was 8 μs. The relatively high stimulation rate was selected to encode the highest target AM frequency (356 Hz) and to approximate the cumulative stimulation rate used in some clinical processors. The spacing between electrodes was varied to represent different amounts of channel interaction; electrodes were either widely (El 4, 10, and 16) or narrowly spaced (EL 9, 10, and 11). The component electrodes of the multi-channel stimuli were optimally interleaved in time; the inter-pulse interval (between the offset of one pulse and the onset of the next) was 0.109 ms. All stimuli were presented via research interface [32], bypassing subjects’ clinical processors and settings; custom software was used to deliver the stimuli and to record subject responses.

Several steps were taken to determine the current levels for the component electrodes in the multi-channel stimuli and to ensure similar loudness across component electrodes and the wide and narrow spacing conditions, and are more fully described in a previous related study [21]. First, the dynamic range (DR) was estimated for single electrodes without AM. Absolute detection thresholds (Ts) were estimated using a “counting” method, as is sometimes used for clinical fitting of speech processors. During each threshold measurement, a number of pulse train bursts (between 2 and 5 bursts) were presented to the subject, who responded by reporting how many bursts were heard. Depending on the correctness of response, the current level was adjusted in 0.5 dB steps; the current level after 4 reversals was considered the threshold. Maximum acceptable loudness (MAL) levels were estimated by slowly increasing the current level (in 0.2 dB steps) for three pulse train bursts until reaching MAL. Threshold and MAL levels were averaged across a minimum of two runs, and the DR was calculated as the difference in current between MAL and T levels. After the initial DR estimation, all electrodes were swept for equal loudness at 10% DR, 50% DR, and at MAL (100% DR). During loudness sweeping, 300 ms pulse trains were delivered to each electrode in sequence (at either 10% DR, 50% DR or MAL, depending on the sweep), first from apex to base, and then from base to apex. The subject indicated which (if any) of the electrodes were louder or softer than the rest; the current level was adjusted to those electrodes as needed, and the electrodes were then re-swept for loudness. After making all adjustments, the final threshold, MAL and DR values for each electrode were recorded.

**Fig 1:**
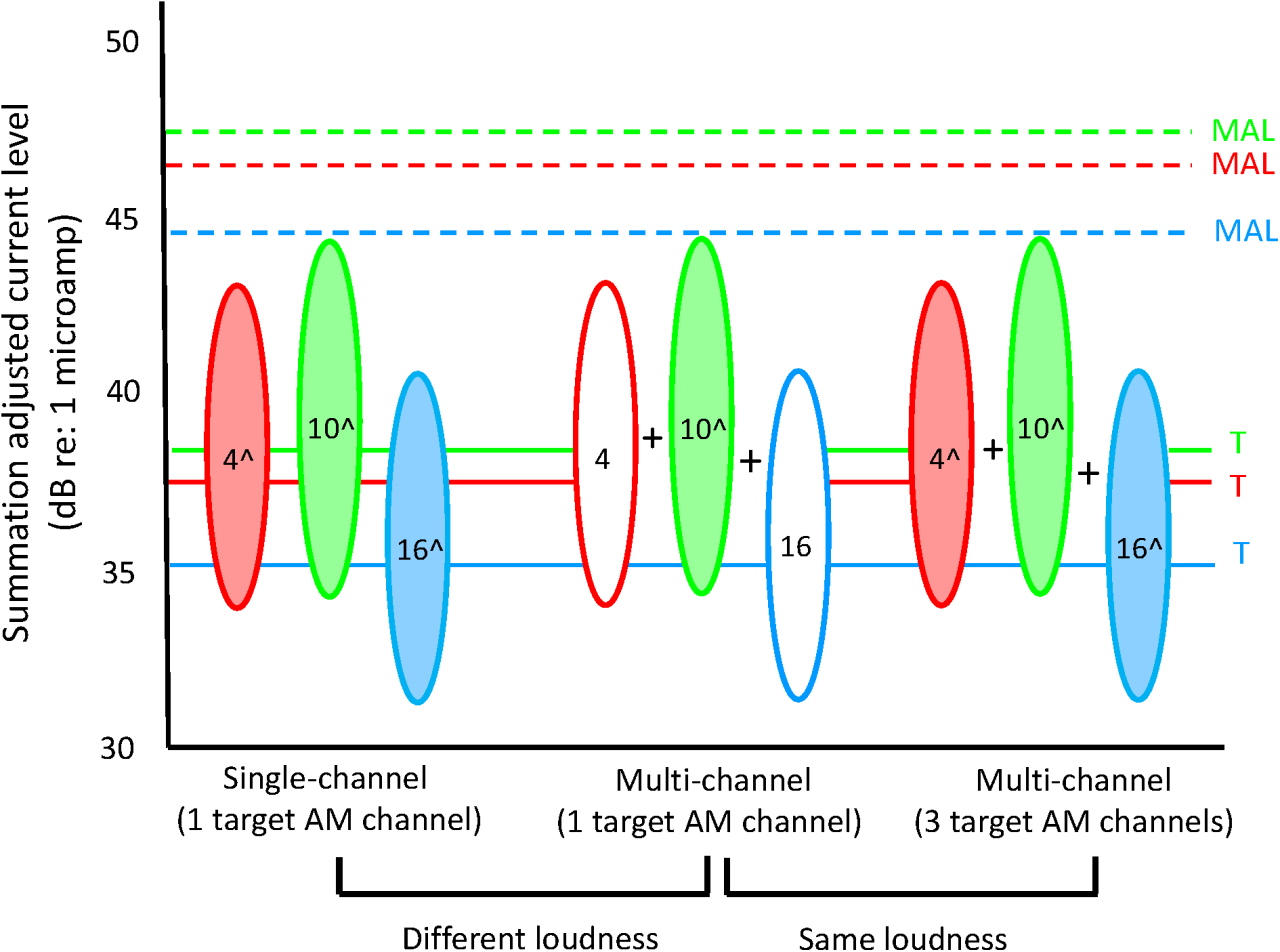
Illustration of the summation-adjusted current levels and DRs used for subject S3. The colored solid and dashed lines show the original single-channel T and MAL levels before adjusting for multi-channel loudness summation, respectively. The ovals represent the summation-adjusted DRs, and also represent the AM depth used to measure AMFD (i.e., between the summation-adjusted T and MAL levels); the number within each oval indicates the electrode. The ovals on the left side of the figure show single-channel stimuli; the ovals in the middle and right side of the figure show multi-channel stimuli. The filled ovals indicate the channels that received the target AM frequency and the white ovals indicate the channels that received the reference AM frequency.

When the three component electrodes were combined using the above single-channel current levels, multi-channel stimulation would be expected to be substantially louder due to summation [17–19]. Multi-channel stimuli were loudness-balanced to a common single-channel reference (EL 10) presented at 50% DR. An adaptive two-alternative, forced-choice (2AFC), double-staircase procedure was used for loudness balancing [33–34]. Stimuli were loudness-balanced without AM. The amplitude of the 3-channel probe was globally adjusted (final step size = 0.4 dB) according to subject response (2-down/1-up or 1-down/2-up, depending on the track), thereby adjusting the current level for each component electrode by the same ratio. For each run, the final 8 of 12 reversals in current level were averaged, and the mean of 2–3 runs was considered to be the loudness-balanced level. The mean current level reduction to the multi-channel stimuli across the wide and narrow combinations was 3.95 dB (range = 1.6 to 6.0 dB), relative to the single-channel reference. Refer to the previous related study [21] for additional details regarding the loudness balance procedure, and for the amount of current level reduction needed to compensate for multi-channel loudness summation for each subject. Fig 1 shows the summation-adjusted DRs for widely spaced electrodes for subject S3. Note that the summation-adjusted current levels were well below the original single-channel T (solid lines) and MAL levels (dashed lines).

Table 2 shows the test electrodes for each subject and condition and the current levels for summation-adjusted T levels (minimum AM current level), MAL levels (maximum AM current level), DR (which corresponds to the range of AM), and 50% DR (which corresponds to the reference current level used to calculate AM depth). Because of the previous loudness sweeping with single electrodes, component electrodes were presumed to be similarly loud at the summation-adjusted T, MAL, and 50% DR current levels. When measuring multi-channel AMFD, the current levels of the component channels were independently roved by ±1 dB from trial to trial to reduce potential cues arising from cross-channel loudness differences.

**Table 2:** Summation-adjusted current levels.

			microamps				dB (re: 1 microamp)			
Subject	Spacing	El	T	MAL	DR	50% DR	T	MAL	DR	50% DR
		4	158	693	535	426	43.97	56.82	12.85	52.58
**S1**	**Wide**	10	155	816	661	485	43.80	58.23	14.44	53.72
		16	126	640	513	383	42.03	56.12	14.09	51.67
		9	163	825	662	494	44.24	58.33	14.09	53.87
**S1**	**Narrow**	10	166	877	711	522	44.42	58.86	14.44	54.34
		11	141	825	684	483	42.98	58.33	15.35	53.67
		4	84	228	144	156	38.48	47.14	8.66	43,85
**S2**	**Wide**	10	70	232	162	151	36.92	47.30	10.38	43.58
		16	71	212	140	142	37.08	46.52	9.44	43.02
		9	69	221	152	145	36.81	46.90	10.09	43.24
**S2**	**Narrow**	10	66	217	151	141	36.35	46.73	10.38	43.01
		11	65	209	145	137	36.20	46.42	10.23	42.74
		4	50	142	92	96	34.02	43.05	9.03	39.66
**S3**	**Wide**	10	54	161	108	107	34.57	44.15	9.58	40.62
		16	38	108	71	73	31.52	40.70	9.18	37.27
		9	47	144	97	95	33.37	43.15	9.79	39.57
**S3**	**Narrow**	10	46	139	93	92	33.26	42.84	9.58	39.31
		11	49	139	89	94	33.88	42.84	8.96	39.47
		4	65	209	145	137	36.20	46.42	10.23	42.73
**S4**	**Wide**	10	50	142	92	96	34.02	43.05	9.03	39.66
		16	54	161	108	107	34.57	44.15	9.58	40.62
		9	111	312	201	212	40.94	49.89	8.95	46.52
**S4**	**Narrow**	10	105	306	201	206	40.46	49.72	9.26	46.27
		11	105	354	249	230	40.46	50.98	10.53	47.22
		4	77	224	147	151	37.76	47.01	9.25	43.56
**S5**	**Wide**	10	82	283	202	183	38.23	49.05	10.82	45.23
		16	71	220	149	145	36.97	46.85	9.88	43.25
		9	68	259	191	164	36.66	48.27	11.61	44.28
**S5**	**Narrow**	10	77	269	191	173	37.76	48.58	10.82	44.76
		11	74	274	199	174	37.44	48.74	11.30	44.81

Values are shown for threshold (T), maximum acceptable loudness (MAL), dynamic range (DR), and 50% DR. The AM depth was between T and MAL (100% DR), and the reference current level was 50% DR.

For the multi-channel stimuli, the basal, middle, and apical channels were sequentially interleaved. Sinusoidal AM was then applied to the multi-channel stimulus according to f(t)*(1+msin(2π*f_m_t)), where f(t) is a steady-state pulse train, m is the modulation index, and f_m_ is the modulation frequency. A 10-ms onset and offset was applied to all stimuli. The initial modulation phase was 180 degrees for all stimuli. For each channel, the modulation index was calculated relative to the reference current level (50% DR, in microamps) to target minimum and maximum current levels at T and MAL, respectively.

**Fig 2:**
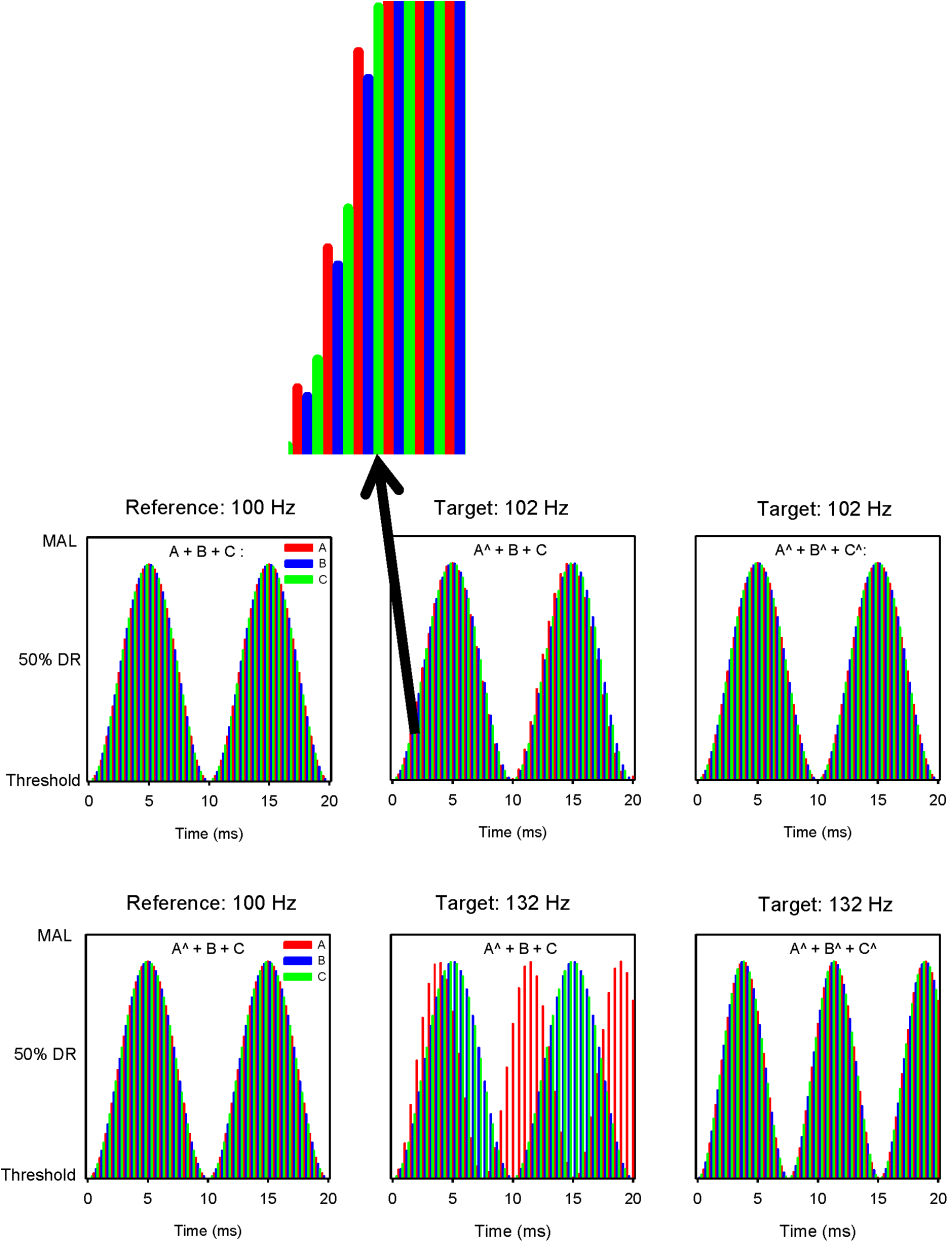
Examples of experimental stimuli. The reference stimuli are shown in the left column and the probe stimuli are shown in the middle and right columns. The top row shows probe stimuli with the 102 Hz target AM frequency and the bottom row shows probe stimuli with the 132 Hz target AM frequency. The left column shows the reference AM frequency delivered to all 3 channels, the middle column shows the target AM frequency delivered to 1 of 3 channels (with the reference AM delivered to the other 2 channels), and the right column shows the target AM frequency delivered to all 3 channels. The x-axis shows time (in ms). The y-axis shows the nominal summation-adjusted current levels. The figure accurately shows the timing of the pulse trains and order of interleaving over a 20 ms range. The close-up of the stimulation pattern shows the current levels for the target AM channel (red) and the reference AM channels (blue and green) for the 102 Hz target AM frequency.

Throughout this paper, the caret symbol (^) indicates the channel that received the target AM. The reference AM frequency was 100 Hz; the target AM frequency was 101, 102, 104, 108, 116, 132, 164, 228, or 356 Hz. During AMFD testing, the reference stimulus contained the reference frequency delivered to all 3 channels. The probe stimulus contained the target AM frequency delivered to one channel and the reference AM frequency delivered to the other two channels. Fig 2 shows examples of the reference and probe stimuli. The envelope patterns are very similar between the 100 Hz reference and the 102 Hz target, but very different between the 100 Hz reference and the 132 Hz target. When the target AM was delivered to only 1 of 3 channels, there is very little difference in the 102 Hz temporal pattern compared to when the target AM was delivered to all 3 channels. However, the difference in the 132 Hz temporal pattern was quite large when the target AM was delivered to 1 of 3 channels or to all 3 channels.

**Fig 3:**
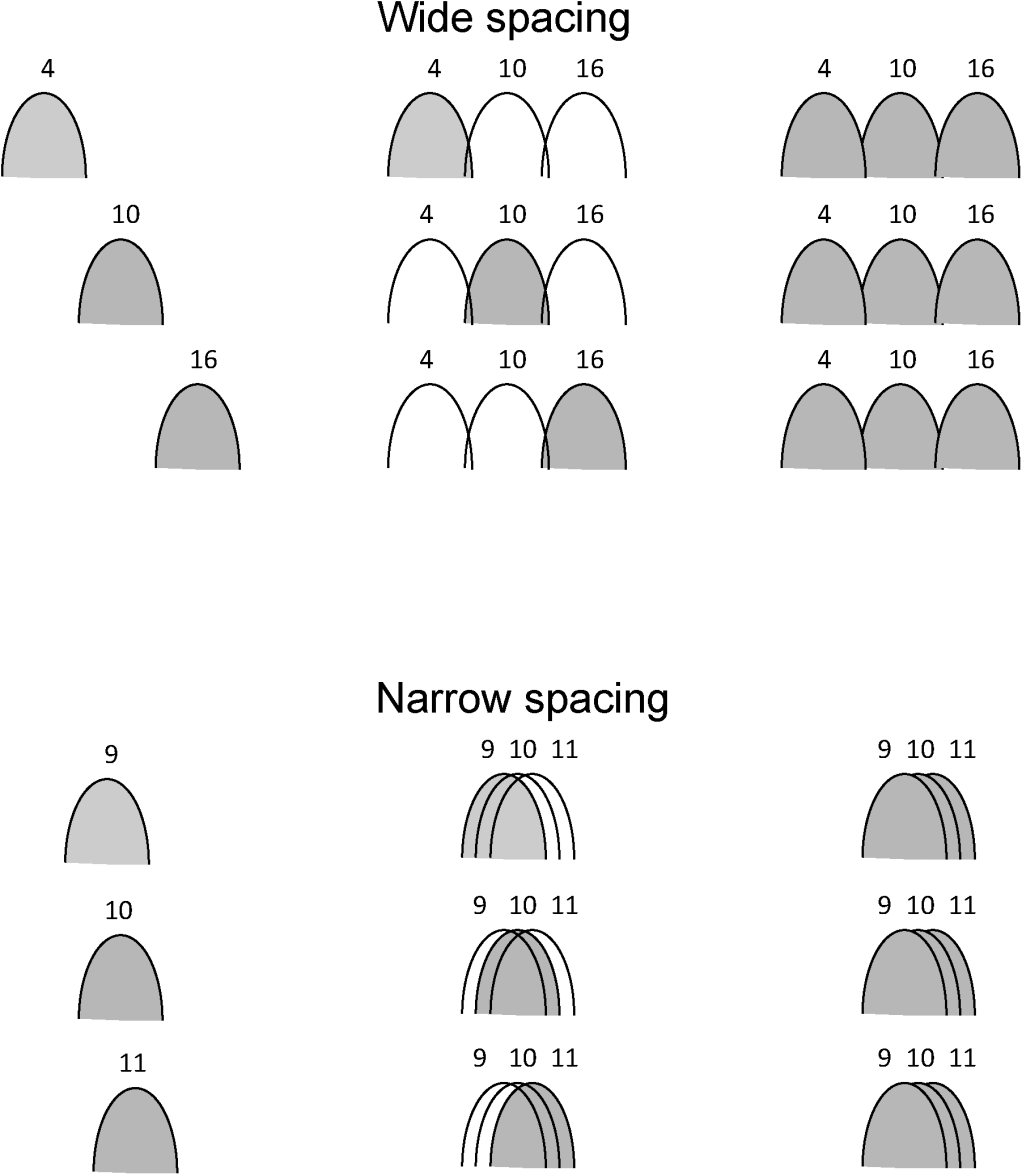
Illustration of electrode spacing conditions. The wide spacing is shown at top and the narrow spacing is shown at bottom. The gray regions indicate the target AM frequency channels and the white regions indicate the reference AM frequency channels. The target AM was delivered to a single channel (left), 1 of 3 channels (middle), or to all 3 channels (right).

Fig 3 illustrates the test conditions in terms of the electrode spacing. For the wide spacing, channels were expected to relatively independent; for the narrow spacing, channels were expected to interact.

### Procedure

A 3AFC non-adaptive procedure was used to measure AMFD (“which interval is different?”), as in a previous related study [21]. During each trial of the run, the probe stimulus (in which the target AM was delivered to 1 channel and the reference AM was delivered to the other 2 channels) was randomly assigned to 1 of the 3 intervals and the reference stimulus (in which the reference AM frequency was delivered to all 3 channels) was assigned to the remaining 2 intervals. The subject was asked to respond which interval was different. Because AM frequency may affect loudness given a fixed AM depth [35], the current level in each interval was globally roved by ± 1 dB to protect against potential loudness differences across AM frequencies as in previous studies [9,21,24,29]. This roving was in addition to the independent roving to current levels across channels in the multi-channel stimuli. Each test run contained 5 reference-probe comparisons for each probe; the reference-probe comparisons were randomized within each run. Three to six test runs were conducted for each condition, depending on subjects’ availability for testing. No trial-by-trial feedback as to the correctness of the response was provided. The test order was randomized within and across subjects.

## Results

**Fig 4:**
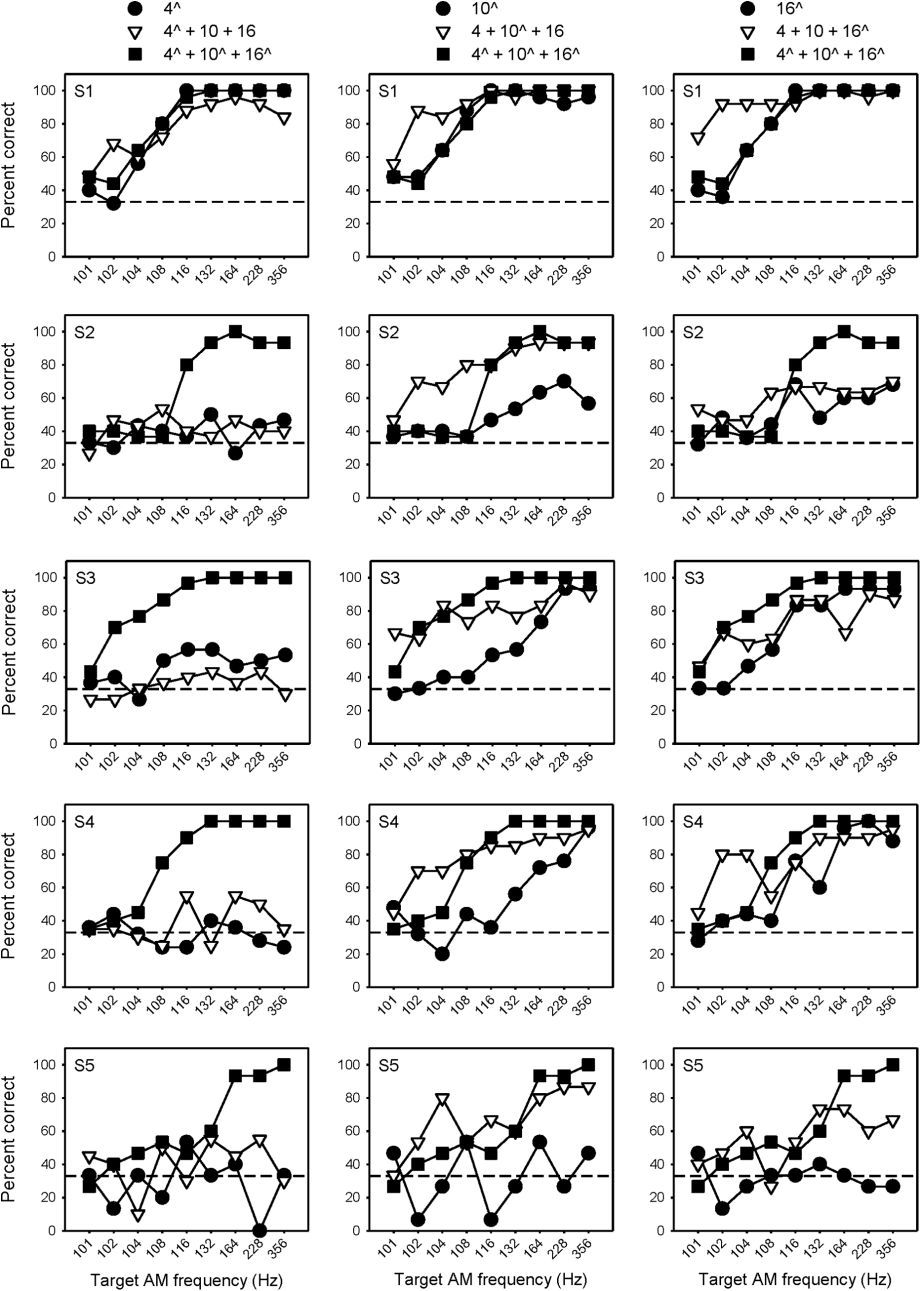
Percent correct AMFD at each probe frequency with wide electrode spacing. Each row shows individual subject data. Each column shows data when the target AM was delivered to only the basal (left), middle (middle), or apical channel (right), or to all 3 channels. The white triangles show data from the present study, in which the target AM was delivered to 1 of 3 channels. The black symbols show data from a related previous study [21], in which the target AM was delivered to a single channel (circles) or to all 3 channels (squares). The caret symbol (^) indicates the target AM channel(s). The dashed line shows chance level performance (33% correct).

Fig 4 shows percent correct AMFD for the wide spacing condition when the target AM was delivered to a single channel (black circles), 1 of 3 channels (white triangles) or to all 3 channels (black squares). The circle and square data are from a previous related study [21], and are shown for comparison purposes. Performance was generally best when the target AM was delivered to all 3 channels. When the target AM was delivered to only a basal channel, with (white triangles) or without the additional 100-Hz reference channels (black circles), performance was generally poor. At relatively low target AM frequencies (102–104 Hz), there were several instances where multi-channel performance was better when the target AM was delivered to the middle or apical channels, rather than to all 3 channels.

**Fig 5:**
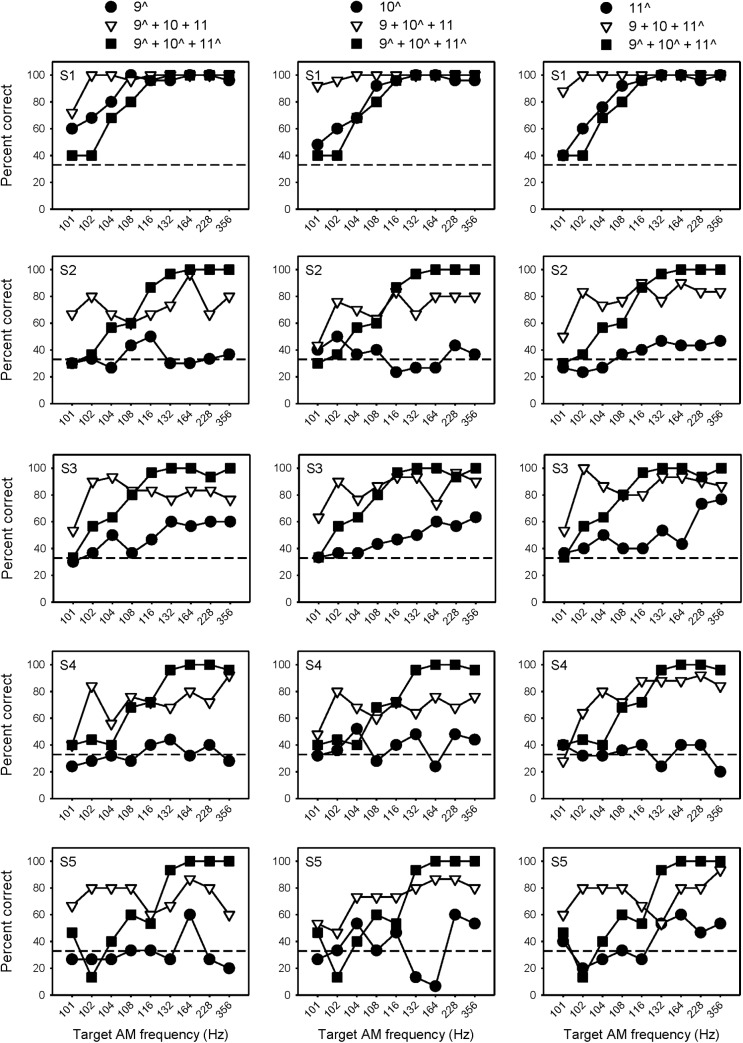
Percent correct AMFD at each probe frequency with narrow electrode spacing. Each row shows individual subject data. Each column shows data when the target AM was delivered to only the basal (left), middle (middle), or apical channel (right), or to all 3 channels. The white triangles show data from the present study, in which the target AM was delivered to 1 of 3 channels. The black symbols show data from a related previous study [21], in which the target AM was delivered to a single channel (circles) or to all 3 channels (squares). The caret symbol (^) indicates the target AM channel(s). The dashed line shows chance level performance (33% correct).

Similarly, Fig 5 shows percent correct AMFD for the narrow spacing condition when the target AM delivered to a single channel, 1 of 3 channels or to all 3 channels; again, the black circle and square data are from a previous related study [21] and are shown for comparison purposes. Different from the wide spacing condition, multi-channel performance was similar when the target AM was delivered to 1 of 3 channels, regardless of target AM channel. At low target AM frequencies (102–104 Hz), performance was markedly better when the target AM was delivered to 1 of 3 channels rather than to all 3 channels. At high target AM frequencies (> 132 Hz), performance tended to be better when the target AM was applied to all 3 channels.

A three-way repeated-measures analysis of variance (RM ANOVA) was performed on the data collected for the present study (i.e., the white triangle data in Figs 4 and 5), with electrode spacing (wide or narrow), target AM channel (relatively basal, middle, or apical), and target AM frequency (101, 102, 104, 108, 116, 132, 164, 228, and 356 Hz) as factors. Results showed no significant main effects of electrode spacing [F(1,7) = 3.345, p = 0.105], target AM channel [F(2,14) = 2.07, p = 0.161], or target AM frequency [F(8,56) = 1.01, p = 0.442]. However, there were significant interactions between electrode spacing and target AM channel [F(2,14) = 7.52, p = 0.006], and between electrode spacing and target AM frequency [F(8,56) = 2.53, p = 0.020]. Because of these interactions, subsequent separate two-way RM ANOVAs were performed on the white triangle data from Figs 4 and 5, with target AM channel and target AM frequency as factors. The results are shown in Table 3. For both the wide and narrow spacing, there were significant effects of target AM channel and target AM frequency (p <0.05 in both cases). For the wide mode, AMFD was significantly poorer when the target AM was delivered to the basal channel (p < 0.05). In many cases, AMFD was significantly poorer with the 101 Hz target AM (p < 0.05).

**Table 3:** Results of the separate two-way RM ANOVAs performed on the white circle data (i.e., the target AM delivered to 1 of 3 channels) shown in Figs 4 and 5.

Spacing	Factor	dF,res	F ratio	*p*	Post-hoc (Bonferroni, p < 0.05)
**Wide**	AM ch	2, 64	22.74	< 0.001	Apical, middle > basal
**Wide**	AM freq	8, 64	23.16	< 0.001	132, 164, 228, 356 > 101, 102, 104, 108;
					102, 104, 108, 116 > 101
**Wide**	AM ch X AM freq	16, 64	2.11	0.018	Middle: 104, 108, 116, 132, 164 > 101
					Apical: 132, 346 > 101, 108; 116, 164, 228 > 101
**Narrow**	AM ch	2, 64	4.75	0.044	
**Narrow**	AM freq	8, 64	12.00	< 0.001	102, 104, 108, 116, 164, 228, 356 > 101
**Narrow**	AM ch X AM freq	16, 64	0.76	0.719	Basal: 102, 164 > 101
					Middle: 116, 164, 228, 356 > 101
					Apical: 102, 104, 108, 116, 132, 164, 228, 356 > 101

AM ch = target AM channel (relatively basal, middle, or apical); AM freq = target AM frequency (101, 102, 104, 108, 116, 132, 164, 228, or 356 Hz); dF, res = degrees of freedom, residual error.

**Fig 6:**
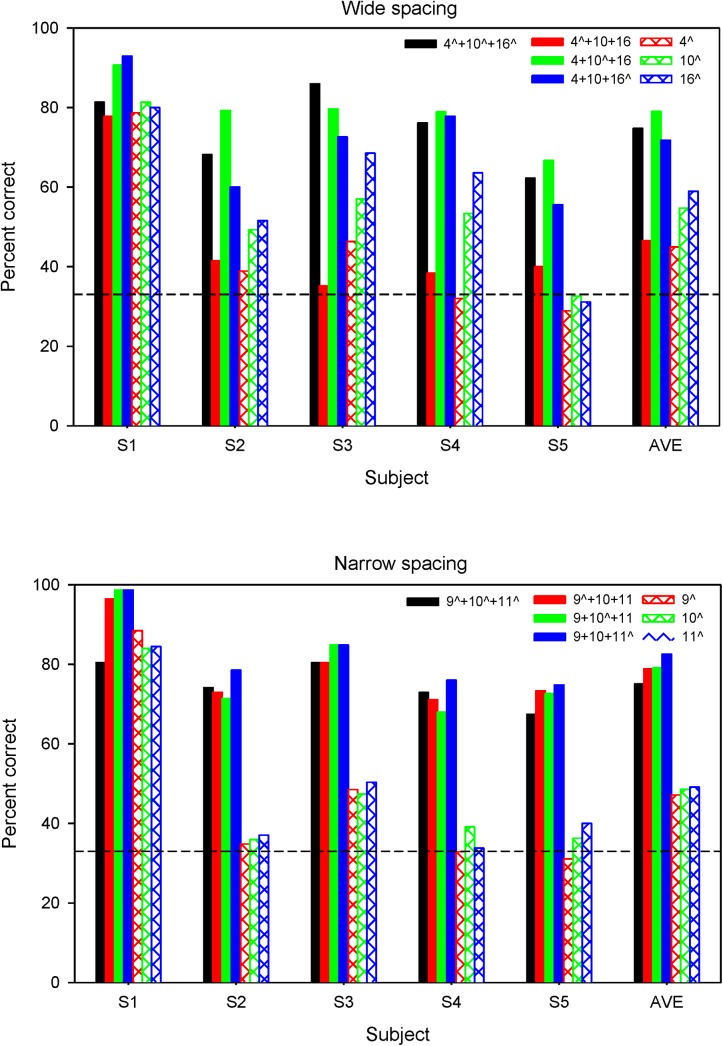
Mean percent correct AMFD across all probe frequencies. Individual and average data is shown. The top and bottom panels show mean AMFD for the wide and narrow spacing, respectively. The black bars show performance when the target AM was delivered to all 3 channels and the hatched colored bars show performance when the target AM was delivered to a single channel; data are from a previous related study [21]. The filled bars show performance when the target AM was delivered to a 1 of 3 channels. The caret symbol (^) indicates the target AM channel(s). The dashed line shows chance-level performance (33.3% correct).

In Figs 4 and 5, AMFD across target AM frequency was often non-monotonic when the target AM was delivered to a single channel (black circles) or to 1 of 3 channels (white triangles). As such, it is difficult to estimate AMFD threshold. Fig 6 shows mean percent correct AMFD (across all target AM frequencies) for the wide and narrow electrode spacing when the target AM was delivered to a single channel, 1 of 3 channels or to all 3 channels. Note that the data when the target AM was presented to a single channel or to all 3 channels are from a previous related study [21] and are presented for comparison purposes. For the wide spacing, mean performance was generally poorer when the target AM was delivered to a single channel (hatched color bars), and poorest when delivered to a single basal channel (hatched red bars). Average performance was similar when the target AM was delivered to a single basal channel (hatched red bars) or to the basal channel with the 100 Hz reference delivered to the apical and middle channel (solid red bars). For the narrow spacing, mean percent correct was near chance-level when the target AM was delivered to any of the single channels (hatched color bars), except for subject S1. Performance sharply improved when the target AM was delivered to 1 of 3 channels or to all 3 channels.

A three-way RM ANOVA, with electrode spacing, target AM channel, and target AM condition (single channel, 1 of 3 channels, or all 3 channels) was performed on the mean AMFD data in Fig 6. Again, note that the data when the target AM was delivered to a single channel or to all 3 channels are from a previous related study [21]. Results showed no significant effects for electrode spacing [F(1,7) = 1.41, p = 0.253], target AM channel [F(2,14) = 1.49, p = 0.259], or target AM condition [F(2,14) = 1.99, p = 0.129]. There were significant interactions between electrode spacing and target AM condition [F(2,14) = 6.41, p = 0.011], between electrode spacing and target AM channel [F(2,14) = 6.34, p = 0.011], between target AM condition and target AM channel [F(4,28) = 6.84, p = 0.001], and among all three factors [F(4,28) = 6.31, p = 0.001]. Because of these interactions, subsequent separate two-way RM ANOVAs were performed for the each target AM condition shown (single-channel, 1 of 3 channels, or all 3 channels) in Fig 6, with electrode spacing and target AM channel as factors. The results are shown in Table 4. When the target AM was delivered to a single channel or to all three channels, there was no significant effect of electrode spacing. When the target AM was delivered to 1 of 3 channels, there were significant effects of electrode spacing and target AM channel (p <0.05), largely driven by the poorer mean AMFD when the target AM was delivered to the basal channel in the wide spacing.

**Table 4:** Results of RM ANOVAs performed on the mean AMFD data shown in Fig 6. Separate analyses were performed for the different target AM channel conditions (i.e., the target AM delivered to a single channel, 1 of 3 channels, or all three channels).

Target AM condition	Factor	dF, res	F-ratio	*p*	Post-hoc (Bonferroni, p < 0.05)
	Spacing	1, 8	1.23	0.330	
**Single channel**	AM ch	2, 8	6.26	0.023	Apical > basal
	Spacing X AM ch	2, 8	3.65	0.075	Wide: apical > basal
	Spacing	1, 8	29.57	0.006	Narrow > Wide
**1 of 3 channels**	AM ch	2, 8	23.85	< 0.001	Apical, middle > basal
	Spacing X AM ch	2, 8	19.34	< 0.001	Wide: apical, middle > basal
					Basal: narrow > wide
**All 3 channels**	Spacing	1, 4	0.02	0.905	

Spacing = wide or narrow, AM ch = target AM channel (relatively basal, middle, or apical); dF, res = degrees of freedom, residual error.

**Fig 7:**
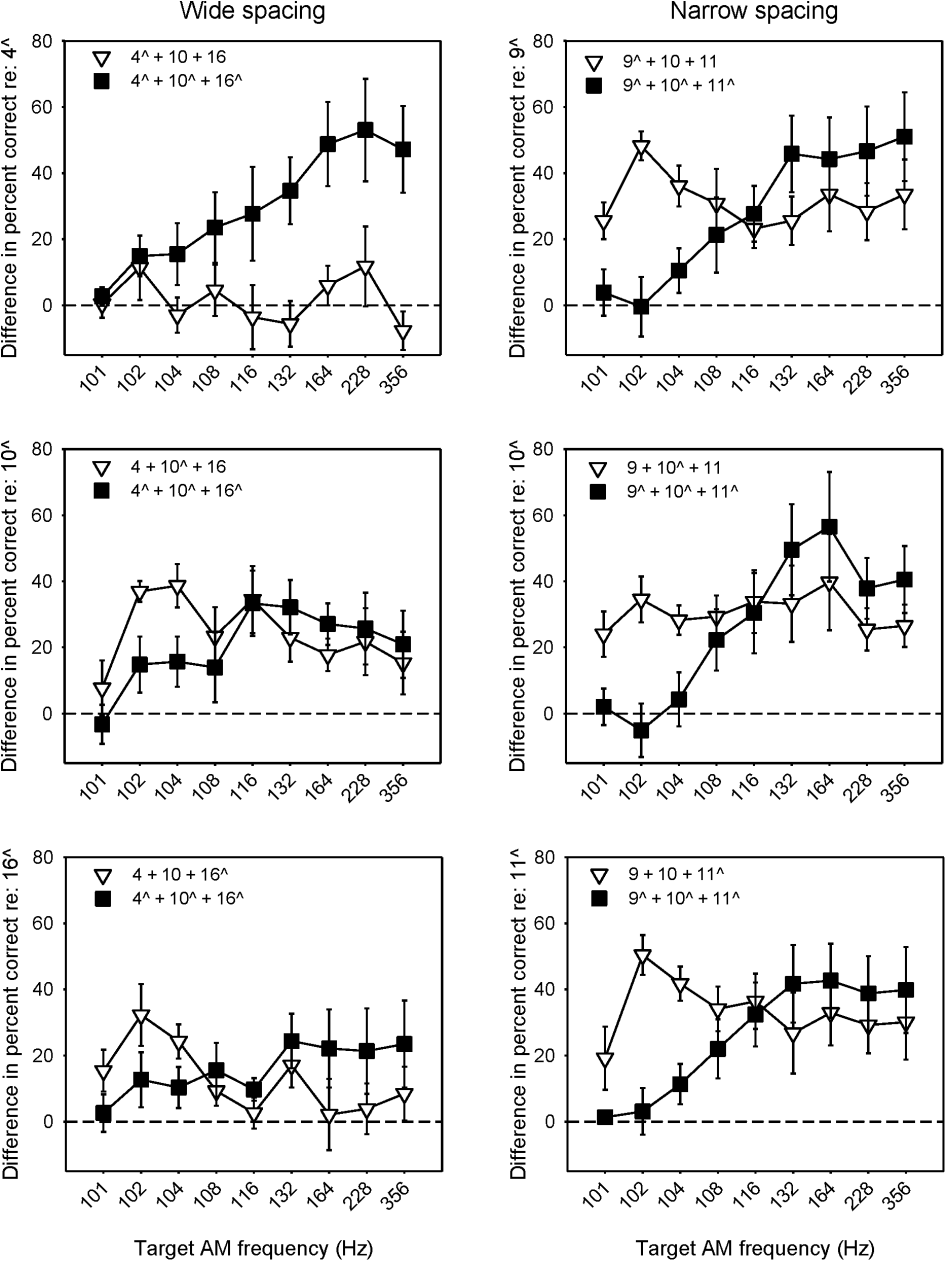
Mean difference in percent correct (across subjects) when the target AM was delivered to 1 of 3 channels or to all 3 channels, relative to a single channel. The left and right columns show data for the wide and narrow spacing conditions, respectively. The top, middle, and bottom rows show data relative to the single basal, middle, or apical channel, respectively. The caret symbol (^) indicates the target AM channel(s). The black squares show the mean difference when 2 target AM channels were added to the single target AM channels; the white triangles show performance when 2 reference AM channels were added to the single target AM channels. The error bars show the standard error. Data for the single-channel reference and when the target AM was delivered to all 3 channels (black squares) were collected in a previous related study [21].

As shown in Figs 4–6, AMFD improved greatly when two channels were added to a single target AM channel, whether with 2 reference or 2 target AM channels. At some target AM frequencies, the improvement in AMFD with multi-channel stimulation was sometimes greater when 2 reference AM channels were added rather than 2 target AM channels. Fig 7 shows the mean difference in percent correct (across subjects) when the target AM was delivered to 1 of 3 channels or to all 3 channels, relative to when the target AM was delivered to a single channel (i.e., the mean difference between the white triangle data and the black circle and square data from Figs 4 and 5). Again, note that the data when AM was delivered to a single channel or to all 3 channels are from a previous related study [21]. Values greater than zero indicate that performance was better with the multi-channel stimuli; values less than zero indicate that performance was better with single-channel stimuli. Note that performance with a single channel (black circle data in Figs 4 and 5) was often quite poor and often near chance level, especially for the narrow spacing. In general, multi-channel performance was better than single-channel performance. One exception was the pattern of results for the multi-channel stimuli relative to single electrode 4 (top left panel of Fig 7). When 2 target AM channels were added, performance sharply improved with AM frequency; there was little effect when 2 reference AM channels were added to single electrode 4. At low target AM frequencies (102–104 Hz), performance was often better when 2 reference AM channels rather than 2 target AM channels were added to the single channel. At higher target AM frequencies (>132 Hz), performance was often better when 2 target AM channels rather than 2 reference AM channels were added to the single channel. In general, there was a near monotonic improvement in performance with target AM frequency when the target AM was delivered to all 3 channels (black squares in Fig 7). When the target AM was delivered to only 1 of 3 channels (white triangles in Fig 7), performance also improved, but without a consistent relationship to target AM frequency.

A four-way RM ANOVA was performed on the difference data shown in Fig 7, with electrode spacing, additional channel type (2 reference AM or 2 target AM), target AM channel, and target AM frequency as factors. Note again that the data for reference single channel data and when 2 target AM channels were added were from a related previous study [21]. The results are shown in Table 5. While there were no significant main effects, there were significant interactions between electrode spacing and additional channel type, additional channel type and target AM channel, additional channel type and target AM frequency, and among electrode spacing, additional channel type, and target AM channel (p < 0.05 in all cases).

**Table 5:** Results from a four-way RM ANOVA performed on the difference data shown in Fig 7.

Factor	dF, res	F-ratio	*p*
Spacing	1, 8	2.77	0.134
Add ch	1, 8	1.12	0.321
AM ch	2, 16	0.96	0.403
AM freq	8, 64	1.01	0.442
Spacing X Add ch	1, 8	6.77	0.032
Spacing X AM ch	2, 16	2.60	0.105
Spacing X AM freq	8, 64	1.34	0.242
Add ch X AM ch	2, 16	7.26	0.006
Add ch X AM freq	8, 64	7.67	< 0.001
AM ch X AM freq	16, 128	1.61	0.074
Spacing X Add ch X AM ch	2, 16	6.83	0.007
Spacing X Add ch X AM freq	8, 64	1.81	0.091
Spacing X Am ch X AM freq	16, 128	1.13	0.334
Add ch X AM ch X AM freq	16, 128	1.62	0.073
Spacing X Add ch X AM ch x AM freq	16, 128	1.23	0.265

Spacing = wide or narrow, Add ch = type of channels added to the single target AM channel (2 reference AM or 2 target AM); AM ch = target AM channel (relatively basal, middle, or apical); AM freq = target AM frequency (101, 102, 104, 108, 116, 132, 164, 228, or 356 Hz); dF, res = degrees of freedom, residual error.

Because of the interaction shown in the previous four-way RM ANOVA, separate two-way RM ANOVAs were performed on the difference data shown in each panel of Fig 7, with added channel type (2 reference or 2 target AM channels) and target AM frequency as factors. The results are shown in Table 6. Adding 2 target AM channels was significantly better than adding 2 reference AM channels only relative to single target AM channel 4 (p < 0.05; top left panel of Fig 7). For the narrow spacing, the difference in AMFD was significantly better when adding 2 reference AM channels than when adding 2 target AM channels, only for 102 and 104 Hz (p < 0.05). Relative to single AM channel 10, the difference in AMFD was significantly greater when adding 2 reference AM channels than when adding 2 target AM channels, for 102 Hz and only for the wide spacing (p < 0.05), Although there appeared to be a greater difference in AMFD when adding 2 target AM channels for frequencies > 132 Hz, there was no significant effect, except relative to single channel 4 (top left panel of Fig 7).

**Table 6:** Results of two-way RM ANOVAs performed on data shown in Fig 7, with the type of added channel and target AM frequency as factors.

Spacing	Single-channel ref	Factor	dF, res	F ratio	*p*	Post-hoc (Bonferroni, p < 0.05)
	4	Added ch	1, 32	12.8	0.023	2 target AM > 2 ref AM
**Wide**	4	AM freq	8, 32	1.7	0.135	
	4	Added ch X AM freq	8, 32	6.3	< 0.001	2 target AM: 164, 228 > 101
						256: 2 target AM > 2 ref AM
	10	Added ch	1, 32	2.0	0.234	
**Wide**	10	AM freq	8, 32	1.7	0.148	
	10	Added ch X AM freq	8, 32	7.1	< 0.001	102: 2 ref AM > 2 target AM
	16	Added ch	1, 32	0.5	0.531	
**Wide**	16	AM freq	8, 32	0.6	0.790	
	16	Added ch X AM freq	8, 32	5.4	< 0.001	
	9	Added ch	1, 32	1.3	0.318	
**Narrow**	9	AM freq	8, 32	2.9	0.014	356 > 101
	9	Added ch X AM freq	8, 32	25.8	< 0.001	2 target AM: 132, 164, 228, 356 >
						101, 102, 104;
						102, 104: 2 ref AM > 2 target AM
	10	Added ch	1, 32	1.0	0.372	
	10	AM freq	8, 32	2.4	0.035	
**Narrow**	10	Added ch X AM freq	8, 32	27.3	< 0.001	2 target AM: 132, 164 > 101, 102, 104;
						356 > 102;
						102: 2 ref AM > 2 target AM
	11	Added ch	1, 32	7.4	0.053	
**Narrow**	11	AM freq	8, 32	1.7	0.133	
	11	Added ch X AM freq	8, 32	15.0	< 0.001	2 target AM: 132, 164 > 101, 102;
						356 > 101
						102, 104: 2 ref AM > 2 target AM

Single-channel ref = single channel reference used to calculate performance difference between single- and multi-channel AMFD scores; Added ch = type of channel added to the single AM channel (2 target AM or 2 reference AM channels); AM freq = target AM frequency (101, 102, 104, 108, 116, 132, 164, 228, or 356 Hz); dF, res = degrees of freedom, residual error.

Another series of separate two-way RM ANOVAs were performed on the data shown in Fig 7, this time with electrode spacing (wide or narrow) and target AM frequency as factors. Data was analyzed separately for conditions when 2 reference AM channels or 2 target AM channels were added to the single AM channel. Data was also analyzed separately for the basal, middle, and apical single-channel references. The results are shown in Table 7. There was a significant effect for electrode spacing only when 2 reference AM channels were added (p < 0.05), with a greater difference for the narrow than for the wide spacing. For the basal and middle single-channel references, there was no significant effect of target AM frequency when 2 reference AM channels were added (p > 0.05). When 2 target AM channels were added, there were significant differences between relatively high and low target AM frequencies (p < 0.05), especially for the narrow combination, but no significant differences between the wide and narrow spacing (p > 0.05). It should be noted that for the analyses presented in Tables 6 and 7, power was sometimes quite low due to the small number of subjects.

**Table 7:** Results of two-way RM ANOVAs performed on data shown in Fig 7, with electrode spacing and target AM frequency as factors.

Added channels	Single-channel ref	Factor	dF, res	F ratio	*p*	Post-hoc (Bonferroni, p < 0.05)
	A	Spacing	1, 32	27.8	0.006	Narrow > Wide
**2 reference AM**	A	AM freq	8, 32	1.4	0.237	
	A	Spacing X AM freq	8, 32	1.0	0.437	
	B	Spacing	1, 32	7,8	0.049	Narrow > Wide
**2 reference AM**	B	AM freq	8, 32	1.4	0.249	
	B	Spacing X AM freq	8, 32	0.9	0.949	
	C	Spacing	1, 32	10,9	0.030	Narrow > Wide
**2 reference AM**	C	AM freq	8, 32	2.5	0.029	
	C	Spacing X AM freq	8, 32	0.9	0.548	
	A	Spacing	1, 32	0.3	0.636	
**2 target AM**	A	AM freq	8, 32	8.1	< 0.001	164, 228, 356 > 101, 102, 104; 132 Hz > 101,102 Hz
	A	Spacing X AM freq	8, 32	0.6	0.619	
	B	Spacing	1, 32	2.7	0.179	
**2 target AM**	B	AM freq	8, 32	6.8	< 0.001	132, 164 > 101, 102, 104;
						116, 228, 356 > 101
	B	Spacing X AM freq	8, 32	2.5	0.032	Narrow: 132, 164 > 101, 102, 104;
						116, 228, 356 > 101
	C	Spacing	1, 32	2.5	0.188	
**2 target AM**	C	AM freq	8, 32	4.1	0.002	132, 164, 256 > 101
	C	Spacing X AM freq	8, 32	15.0	< 0.001	Narrow: 132 > 101;
						164 > 101, 102

Single channel ref = single channel used to calculate performance differences between single- and multi-channel AMFD scores; Spacing = wide or narrow; AM freq = target AM frequency (101, 102, 104, 108, 116, 132, 164, 228, or 356 Hz); dF, res = degrees of freedom, residual error.

## Discussion

The present data showed substantial envelope interaction that was not consistently related to difference in AM frequency between the target and reference AM channels. The envelope interference was greater when channels were narrowly spaced than when widely spaced, although there was substantial interference even among widely spaced channels. Below, we discuss the present results in greater detail.

### Envelope interactions

When the target AM was delivered to only 1 of 3 channels, there was not a consistent relationship between AMFD and the differences in AM frequency across multiple channels. However, CI subjects were very sensitive to the presence of envelope interactions at all target AM frequencies. This result is somewhat consistent with previous studies that showed sometimes highly elevated AMFD thresholds when a single masker AM channel was combined with a single target AM channel [28–29].

CI subjects were extremely sensitive to very small AM frequency differences (2–4 Hz) between component channels, especially when channels were narrowly spaced. For the 20 ms segments shown in the top row of Fig 2, the overall temporal envelope appears to be quite similar whether the 102 Hz target AM was delivered to 1 of 3 channels (middle panel) or to all 3 channels. However, given the 300-ms stimulus duration, the 100-Hz reference and 102-Hz target AM would have been out of phase, which may have provided a strong perceptual cue. The bottom row of Fig 2 shows strong differences between the target and reference channels when the 132 Hz target AM was delivered to 1 of 3 channels. Yet performance was quite similar between the 102 and 132 Hz target AM frequencies when the target AM was delivered to 1 of 3 channels (see Figs 4 and 5). Because the present multi-channel stimuli were interleaved in time, and because the interference was greater for narrowly spaced channels, CI subjects may have attended to interactions between envelopes at the neural level (rather than the envelopes themselves interacting). Given the relatively high stimulation rate (2000 pps/channel), neurons responding to stimulation from one channel might not have fully recovered before receiving stimulation from the second and third channels. As such, the temporal envelopes from each channel may have been combined in the overlapping neural region. When the difference in AM rate across channels was small, this may have produced some irregularity in the probe stimuli. Such percepts associated with low AM rate differences were also observed in previous AMFD studies with interferers [36–37]. In this study, this percept persisted for larger AM frequency differences. As such, the present results do not reflect CI subjects’ ability to discriminate target AM frequency in the presence of competing AM channels, but rather their sensitivity to envelope interactions that did not strongly depend on AM frequency differences. This sensitivity may have been somewhat elevated for low AM rate differences between the target and reference AM channels.

The present data showed greater interference among narrowly spaced channels than widely spaced channels, similar to previous studies [28–29]. Still, there was significant interference among widely spaced channels. The spread of excitation might be expected to be reduced given the low summation-adjusted stimulation levels on each channel. The present data suggests that interactions most likely occurred where these channels overlapped, which would have been more pronounced with narrow spacing. In the previous related study [21], there was no significant effect of electrode spacing when the target AM was applied to all 3 channels. When the target AM was applied to only 1 of 3 channels, there was a significant effect of electrode spacing, suggesting that the effect of channel interaction may depend on the type of envelope information delivered to each channel. When the envelope information was the same on all channels, the degree of channel interaction had little effect. When the envelope information was different across channels, the degree of channel interaction mattered greatly. Thus the peripheral pattern may matter more when processing competing rather than coherent envelopes.

The present data also suggests that using adaptive procedures to measure AMFD with interferers may not produce meaningful threshold data. In this study, there was no monotonic relationship between the target AM frequency and performance when the target AM was delivered to 1 of 3 channels. As such, AMFD thresholds derived from an adaptive pitch ranking procedure, as used in some previous studies [28–29] may not accurately reflect perception of frequency differences between component AM channels. Also, adaptive procedures in an AMFD task may not test very low frequency differences between the reference and probe AM rates, as thresholds often are 10% or more of the reference rate [29]. A non-adaptive procedure as used in the present study allows the psychometric function to be directly measured. As such, any non-monotonicities in the psychometric function may be observed. In the present results, the non-adaptive procedure revealed non-monotonic pattern of results when the target AM was delivered to 1 of 3 channels. Given the present pattern of results, it is unclear whether the sometimes greatly elevated thresholds reported in previous AMFD studies [28–29] fully reflect CI users’ ability to perceive target AM rates in the presence of interferers. Even lower thresholds reported for some masked conditions may not reflect the nature of the envelope interactions [28–29], as the present data suggest a dip in the masked threshold function when the target AM frequency difference was between 8 and 32 Hz. The present data suggest substantial interference even when the difference between the target and reference channel AM rates was quite small, and that this interference persisted even when the difference in AM rates was nearly 2 octaves, especially for the narrowly spaced electrodes.

### Multi-channel loudness summation and temporal envelope processing

In a previous related study [21], single-channel AMFD with summation-adjusted current levels was quite poor (see circle data in Figs 4 and 5). In that study, increasing the current of a single channel or adding channels with coherent AM greatly improved performance. In this study, adding channels with different AM to the target AM was easily perceived, though none of the single channels could convey temporal envelope information when presented in isolation. In both cases, there was greater temporal envelope sensitivity with multiple channels. In the previous study [21], the multi-channel advantage was explained by the increased loudness rather than by multiple representations of the temporal envelope. However, the present data suggest that envelope information may have been combined across channels. Loudness summation may still play a role in multi-channel envelope processing, as envelopes may not be effectively combined across channels until achieving some criterion loudness (e.g., comfortably loud). Thus, the present results also support previous work [21,25] in which the multi-channel advantage in AMFD was explained by the multiple representations of envelope information.

One exception to the present pattern of results is subject S1 (top row in Figs 4 and 5). Subject S1 experienced the least amount of multi-channel loudness summation. Consequently, single-channel and multi-channel AMFD were very similar [21]. Note that S1 was also the most sensitive to envelope interactions, exhibiting the highest scores of all subjects at all target AM frequencies. Subject did not have the largest DRs or lowest T levels (see Table 2), so absolute current levels do not explain the greater sensitivity to envelope interactions. Subject S1 exhibited similar effects of electrode spacing as the other subjects, so it is unlikely that there was markedly different channel interaction.

### Contributions of individual channels to multi-channel envelope processing

In the previous related study [21], it was difficult to observe across-site differences in single-channel AMFD. At the summation-adjusted levels, performance was too poor and at comfortably loud levels, performance was too good. As such, contributions of individual channels to the multi-channel percept could not be observed when coherent AM was delivered to all 3 channels. One motivation for the present study was to vary the stimulation site of the target AM channel when the target was delivered to 1 of 3 channels. Across-site differences in this manipulation might reveal channels that strongly or weakly interacted with the others. Channels with better temporal processing might be more resistant to the interferers. Alternatively, channels with poorer temporal processing might interact weakly with channels with better temporal processing.

In the wide spacing, there was little interaction when the target AM was delivered to EL 4 and the reference AM was delivered to EL 10 and EL 16. Indeed, performance was quite similar when the target AM was delivered to EL 4 (circle and triangle data in left column of Fig 4), whether or not the reference AM was delivered to EL 10 and EL 16. Given that AMFD was generally poorest when the target AM was delivered to single EL 4, it is unlikely that good temporal processing made EL 4 more resistant to the interferers. Interestingly, when the target AM was delivered to EL 10 and EL 16 (square data in left column of Fig 4), performance sharply improved. Taken together, these patterns of results suggest that performance was largely driven by EL 10 and EL 16, whether or not coherent AM was delivered to the additional channels. It seems likely that for these subjects and stimuli, temporal processing was poor for EL 4 and thus contributed weakly to multi-channel envelope processing. Such an observation is consistent with previous studies that have suggested better temporal processing in the apical region of the cochlea [38–39], although no strong or consistent advantage has been shown for apical electrodes [40–41]. This result is not consistent with previous studies that have shown no significant effect of interferer location on AMFD [27–28]. Note that in these studies, only 2 channels were stimulated (1 target and 1 interferer), and the target AM was typically delivered to an electrode in the middle of the array. In this study, the stimulation site of the target AM channel was varied across all 3 channels, which may have revealed the different pattern of results.

In the wide spacing, when the target AM was delivered to EL 10 or 16 (middle and bottom left panels of Fig 7, respectively), there was substantial interaction, especially for low target AM frequencies. Interestingly, the largest interaction was observed when the target AM was delivered to EL 10. It is unclear whether this indicates better temporal processing on EL 10 (which might give rise to stronger interaction) or interactions with the spread of excitation from both EL 4 and EL 16. When the target AM was delivered to EL 4 or 16, either would have primarily interacted with EL 10, as the spread of excitation from the most spatially remote electrode would have produced much less interference.

There was a significant interaction between electrode spacing and target AM channel for mean AMFD when the target AM was delivered to only 1 of 3 channels (see Table 4). With the wide spacing, mean AMFD was significantly better when the target AM was delivered to the apical or middle channels, rather than the basal channel. With the narrow spacing, there was no significant difference among target AM channels, most likely because of the strong overlap in the spread of excitation among ELs 9, 10, and 11.

### Limitations to the present study

A 3AFC task was used in this study to measure AMFD, similar to many previous studies [9,13–16,21], rather than a 2AFC pitch ranking task [11,24,25,29]. CI subjects were very sensitive to the channel interactions in this study. As discussed above, a 2AFC adaptive procedure may not be appropriate given the present non-monotonic functions when AMFD was measured with interferers. One alternative would be to measure pitch ranking with interferers using a 2AFC non-adaptive procedure.

The AM depth used in this study was much deeper than typically used in previous AMFD or MDI studies, which is typically some value above MDT [9]. This large AM depth may have contributed to the present pattern of results. It is unclear whether the present pattern of results would hold with smaller AM depths. Also, the summation-adjusted current levels used in this study were quite low, providing very poor single-channel AMFD. Most previous studies have measured AMFD or MDI at higher loudness levels [25,28–28], which provides good AMFD even with a smaller AM depth than used in this study. However, the present summation-adjusted current levels are likely to be more comparable to those used in clinical processors. AMFD measured at these summation-adjusted levels may be more representative of the temporal processing limits within each channel. With multi-channel stimulation, AMFD greatly improves due to increased loudness and/or combined coherent AM information. Unfortunately, channels with different envelope information interact as well, resulting in poor perception of the target AM.

### Implications for CI users

The present results demonstrate the importance of reducing channel interaction in CIs. Envelope interference was reduced in the present wide spacing, relative to the narrow spacing. Results from the previous studies suggest that CI users may benefit from redundant envelope cues presented on multiple channels. As such, similar envelope cues could be delivered to adjacent channels while dissimilar envelope cues could be delivered to spatially remote channels; in such an approach, adequate and accurate representation of the spectral envelope should still be maintained. High rates may further increase channel interaction [42]. As such, lower stimulation rates may improve channel independence and reduce envelope interference. Finally, given the effects of loudness summation on multi-channel envelope processing, it might be advisable to stimulate fewer channels per stimulation cycle. Fewer channels in each cycle may require higher current levels to maintain adequate loudness. The higher current levels may in turn improve temporal processing for each channel and subsequently improve multi-channel envelope perception.

## Conclusions

In this study, multi-channel AMFD was measured using stimuli in which the target AM was delivered to 1 channel and the reference AM was delivered to 2 channels. The spacing between electrodes was varied to be wide or narrow, thereby testing the effect of relative channel interaction on multi-channel AMFD. The stimulation site of the target AM channel was varied to test single-channel contributions to the multi-channel AMFD. The present data were compared to data from a previous study in which the target AM was delivered to a single channel or to all 3 channels; in all cases, AMFD was measured using reduced current levels on each channel to accommodate multi-channel loudness summation. Key findings include:

1. CI subjects were very sensitive to multi-channel envelope interference, especially when electrodes were narrowly spaced.
2. When only the target AM was delivered to 1 of 3 channels, there was not a consistent relationship with target AM frequency. The non-monotonic functions suggest that a non-adaptive procedure, as used in this study may be more appropriate than adaptive pitch ranking tasks used in previous studies that measured AMFD with interfering envelopes.
3. When electrodes were widely spaced, there was little interaction among channels when the target AM was delivered to the most basal channel, possibly due to poorer temporal processing in the basal electrode. The most envelope interaction was observed when the target AM was delivered to the middle electrode and the reference AM was delivered to the apical and basal electrodes, which may have maximized interactions at the edges of the spread of excitation.
4. Data from the previous study [21] showed that single-channel AMFD was very poor at summation-adjusted current levels. When multiple channels were added that contained coherent AM, AMFD improved greatly. When multiple channels were added that contained different AM from the target, CI subjects were very sensitive to envelope interactions. Thus, channels that were not capable of transmitting envelope cues could be combined to deliver envelope information that was easily perceived. This suggests that listeners combined envelope information across channels, in addition to benefitting from the increased loudness associated with multi-channel summation.

## Supporting Information

S1 Table Raw data collected for this study. Each cell shows the number of times (out of 5) that the stimulus was correctly discriminated. Each row shows a test run. Each column shows the target AM frequency. Each tab shows data for individual subjects.
(XLSX)

## References

[1] Donaldson GS, Viemeister NF. Intensity discrimination and detection of amplitude modulation in electric hearing. J Acoust Soc Am; 2000; 108: 760–763. 10955643 10.1121/1.429609

[2] Chatterjee M, Robert ME. Noise enhances modulation sensitivity in cochlear implant listeners: stochastic resonance in a prosthetic sensory system? J Assoc Res Otolaryngol; 2001; 2: 159–171. 11550525 10.1007/s101620010079 PMC3201182

[3] Fu QJ. Temporal processing and speech recognition in cochlear implant users Neuroreport; 2002; 13: 1635–1640. 10.1097/00001756-200209160-00013 12352617

[4] Chatterjee M, Oba SI. Noise improves modulation detection by cochlear implant listeners at moderate carrier levels. J Acoust Soc Am; 2005; 118: 993–1002. 16158655 10.1121/1.1929258

[5] Galvin JJ 3rd, Fu QJ. Effects of stimulation rate, mode and level on modulation detection by cochlear implant users. J Assoc Res Otolaryngol; 2005; 6: 269–279. 16075190 10.1007/s10162-005-0007-6 PMC2504596

[6] Pfingst BE, Xu L, Thompson CS. Effects of carrier pulse rate and stimulation site on modulation detection by subjects with cochlear implants. J Acoust Soc Am; 2007; 121: 2236–2246. 17471737 10.1121/1.2537501 PMC2562216

[7] Galvin JJ 3rd, Fu QJ. Influence of stimulation rate and loudness growth on modulation detection and intensity discrimination in cochlear implant users. Hear Res; 2009; 250: 46–54. 10.1016/j.heares.2009.01.009 19450432 PMC5844469

[8] Chatterjee M, Yu J. A relation between electrode discrimination and amplitude modulation detection by cochlear implant listeners. J Acoust Soc Am; 2010; 127:415–426. 10.1121/1.3257591 20058987 PMC2821169

[9] Chatterjee M, Oberzut C. Detection and rate discrimination of amplitude modulation in electrical hearing. J Acoust Soc Am; 2011; 130:1567–1580. 10.1121/1.3621445 21895095 PMC3188971

[10] Fraser M, McKay CM. Temporal modulation transfer functions in cochlear implantees using a method that limits overall loudness cues. Hear Res; 2012; 283: 59–69. 10.1016/j.heares.2011.11.009 22146425 PMC3314947

[11] Green T, Faulkner A, Rosen S. Variations in carrier pulse rate and the perception of amplitude modulation in cochlear implant users. Ear Hear; 2012; 33: 221–230. 10.1097/AUD.0b013e318230fff8 22367093

[12] Cazals Y, Pelizzone M, Saudan O, Boex C. Low-pass filtering in amplitude modulation detection associated with vowel and consonant identification in subjects with cochlear implants. J Acoust Soc Am; 1994; 96: 2048–2054. 7963020 10.1121/1.410146

[13] Chatterjee M, Peng SC. Processing F0 with cochlear implants: Modulation frequency discrimination and speech intonation recognition. Hear Res; 2008; 235: 143–156. 18093766 10.1016/j.heares.2007.11.004 PMC2237883

[14] Deroche M., Zion DJ, Schurman JR, Chatterjee M. Sensitivity of school-aged children to pitch-related cues. J Acoust Soc Am; 2012; 131: 2938–2947. 10.1121/1.3692230 22501071 PMC3339501

[15] Deroche ML, Lu HP, Limb CJ, Lin YS, Chatterjee M. Deficits in the pitch sensitivity of cochlear-implanted children speaking English or Mandarin. Front. Neurosci. 2014. 10.3389/fnins.2014.00282 PMC4158799 25249932

[16] Luo X, Fu QJ, Wei CG, Cao K. Speech recognition and temporal amplitude modulation processing by Mandarin-speaking cochlear implant users. Ear Hear; 2008; 29: 957–970. 10.1097/AUD.0b013e3181888f61 18818548 PMC2704892

[17] McKay CM, Remine MD, McDermott HJ. Loudness summation for pulsatile electrical stimulation of the cochlea: effects of rate, electrode separation, level, and mode of stimulation. J Acoust Soc Am; 2001; 110: 1514–1524. 11572362 10.1121/1.1394222

[18] McKay CM, Henshall KR, Farrell RJ, McDermott HJ. A practical method of predicting the loudness of complex electrical stimuli. J Acoust Soc Am; 2003; 113: 2054–2063. 12703716 10.1121/1.1558378

[19] Drennan WR, Pfingst BE. Current-level discrimination in the context of interleaved, multichannel stimulation in cochlear implants: effects of number of stimulated electrodes, pulse rate, and electrode separation. J Assoc Res Otolaryngol; 2006; 7: 308–316. 16794913 10.1007/s10162-006-0045-8 PMC2430008

[20] Galvin JJ 3rd, Oba S, Fu QJ, Başkent D. Single- and multi-channel modulation detection in cochlear implant users. PLoS One; 2014; 11; 96:e99338. 10.1371/journal.pone.0099338 PMC4053447 24918605

[21] Galvin JJ 3rd, Oba S, Başkent D, Fu QJ. Modulation frequency discrimination with single and multiple channels in cochlear implant users. Hear Res; 2015; 324:7–18. 10.1016/j.heares.2015.02.007 25746914 PMC4405494

[22] McKay CM, Henshall KR. Amplitude modulation and loudness in cochlear implantees. J Assoc Res Otolaryngol; 2010; 11: 101–111. 10.1007/s10162-009-0188-5 19798533 PMC2820208

[23] Galvin JJ 3rd, Fu QJ, Oba S, Başkent D. A method to dynamically control unwanted loudness cues when measuring amplitude modulation detection in cochlear implant users. J Neurosci Methods; 2014; 222: 207–212. 10.1016/j.jneumeth.2013.10.016 24269251 PMC3897474

[24] Kreft HA, Oxenham AJ, Nelson DA. Modulation rate discrimination using half-wave rectified and sinusoidally amplitude modulated stimuli in cochlear-implant users. J Acoust Soc Am; 2010; 127: 656–659. 10.1121/1.3282947 20136187 PMC2830260

[25] Geurts L, Wouters J. Coding of the fundamental frequency in continuous interleaved sampling processors for cochlear implants. J Acoust Soc Am; 2001; 109: 713–726. 11248975 10.1121/1.1340650

[26] Chatterjee M. Modulation masking in cochlear implant listeners: envelope versus tonotopic components. J Acoust Soc Am; 2003; 113:2042–2053. 12703715 10.1121/1.1555613

[27] Chatterjee M, Oba SI. Across- and within-channel envelope interactions in cochlear implant listeners. J Assoc Res Otolaryngol; 2004; 5:360–375. 15675001 10.1007/s10162-004-4050-5 PMC2504569

[28] Chatterjee M, Oberzut C. Multi-channel interactions in amplitude modulation detection and discrimination by cochlear implant listeners. Conference on Auditory Implants and Prostheses; 2009; 218.

[29] Kreft HA, Nelson DA, Oxenham AJ. Modulation frequency discrimination with modulated and unmodulated interference in normal hearing and in cochlear-implant users. J Assoc Res Otolaryngol; 2013; 14: 591–601. 10.1007/s10162-013-0391-2 23632651 PMC3705089

[30] Chatterjee M, Shannon RV. Forward masked excitation patterns in multielectrode electrical stimulation. J Acoust Soc Am; 1998; 103:2565–2572. 9604350 10.1121/1.422777

[31] Chatterjee M, Galvin JJ 3rd, Fu QJ, Shannon RV. Effects of stimulation mode, level and location on forward-masked excitation patterns in cochlear implant patients. J Assoc Res Otolaryngol; 2006; 7:15–25. 16270234 10.1007/s10162-005-0019-2 PMC2504584

[32] Wygonski J, Robert M. House Ear Institute Nucleus Research Interface (HEINRI) specification—internal materials. 2002.

[33] Jesteadt W. An adaptive procedure for subjective judgments. Percept. Psychophys; 1980; 28: 85–88. 7413416 10.3758/bf03204321

[34] Zeng FG, Turner CW. Binaural loudness matches in unilaterally impaired listeners Quarterly J. Exp. Psych; 1991; 43: 565–583. 10.1080/14640749108400987 1775657

[35] Vandali A, Sly D, Cowan R, van Hoesel R. Pitch and loudness matching of unmodulated and modulated stimuli in cochlear implantees. Hear Res; 2013; 302: 32–49. 10.1016/j.heares.2013.05.004 23685148

[36] Chatterjee M, Oberzut C. Multi-channel interactions in amplitude modulation detection and discrimination by cochlear implant listeners. *2009 Conference on Implantable Auditory Prostheses*, *Lake Tahoe*, *CA;* 2009; 218.

[37] Chatterjee M, Kulkarni A. AM rate discrimination by cochlear implant listeners in on- and off-channel, modulated masking. *2015 Midwinter Meeting of the Association for Research in Otolaryngology*, *Baltimore*, *MD;* 2015; 152.

[38] Middlebrooks JC, Snyder RL. Selective electrical stimulation of the auditory nerve activates a pathway specialized for high temporal acuity. J Neurosci; 2010; 30:1937–1946. 10.1523/JNEUROSCI.4949-09.2010 20130202 PMC2828779

[39] Macherey O, Deeks JM, Carlyon RP. Extending the limits of place and temporal pitch perception in cochlear implant users. J. Assoc. Res. Otolaryngol; 2011; 12: 233–251. 10.1007/s10162-010-0248-x 21116672 PMC3046333

[40] Baumann U, Nobbe A. Pulse rate discrimination with deeply inserted electrode arrays. Hear Res; 2004; 196: 49–57. 15464301 10.1016/j.heares.2004.06.008

[41] Carlyon RP, Lynch C, Deeks JM. Effect of stimulus level and place of stimulation on temporal pitch perception by cochlear implant users. J Acoust Soc Am; 2010; 127: 2997–3008. 10.1121/1.3372711 21117749

[42] Middlebrooks JC. Effects of cochlear-implant pulse rate and inter-channel timing on channel interactions and thresholds. J Acoust Soc Am; 2004; 116:452–468. 15296005 10.1121/1.1760795

